# MicroRNA-7: expression and function in brain physiological and pathological processes

**DOI:** 10.1186/s13578-020-00436-w

**Published:** 2020-06-10

**Authors:** Juanjuan Zhao, Ya Zhou, Mengmeng Guo, Dongxu Yue, Chao Chen, Guiyou Liang, Lin Xu

**Affiliations:** 1 School of Medicine, Guizhou University, Guiyang, 550025 Guizhou China; 2 Department of Medical Physics, Zunyi Medical University, Zunyi, 563000 Guizhou China; 3 Department of Immunology, Zunyi Medical University, Zunyi, 563000 Guizhou China; 4 Specific Key Laboratory of Gene Detection and Treatment of Guizhou Province, Zunyi, 563000 Guizhou China; 5 Department of Cardiovascular Surgery, Affiliated Hospital of Guizhou Medical University, Guiyang, 550004 Guizhou China; 6 Department of Cardiovascular Surgery, Affiliated Hospital of Zunyi Medical University, Zunyi, 563000 Guizhou China

**Keywords:** MiR-7, Expression regulation, Physiological function, Brain diseases

## Abstract

MicroRNAs (miRNAs) are a class of small non-coding RNAs that regulate gene expression at the post-transcriptional level and play critical roles in regulating physiological function, and are becoming worldwide research hot spot in brain development and diseases. However, the exact value of miRNAs in brain physiological and pathological processes remain to be fully elucidated, which is vital for the application of miRNAs as diagnostic, prognostic, and therapeutic biomarkers for brain diseases. MicroRNA-7 (miR-7), as a highly expressed miRNA molecule in the mammalian brain, is well documented to play a critical role in development of various diseases. Importantly, accumulating evidence has shown that miR-7 is involved in a range of developmental and pathological processes of brain. Expressively, miR-7, encoded by three genes located different chromosomes, is dominantly expressed in neurons with sensory or neurosecretory. Moreover, the expression of miR-7 is regulated at three levels including gene transcription, process of primary and precursor sequence and formation of mature sequence. Physiologically, miR-7 principally governs the physiological development of Pituitary gland, Optic nervous system and Cerebral cortex. Pathologically, miR-7 can regulate multiple genes thereby manipulating the process of various brain diseases including neurodegenerative diseases, neuroinflammation, and mental disorders and so on. These emerging studies have shown that miR-7, a representative member of miRNA family, might be a novel intrinsic regulatory molecule involved in the physiological and pathological process of brain. Therefore, in-depth studies on the role of miR-7 in brain physiology and pathology undoubtedly not only provide a light on the roles of miRNAs in brain development and diseases, but also are much helpful for ultimate development of therapeutic strategies against brain diseases. In this review, we provide an overview of current scientific knowledge regarding the expression and function of miR-7 in development and disease of brain and raise many issues involved in the relationship between miR-7 and brain physiological and pathological processes.

## Introduction

**Fig. 1:**
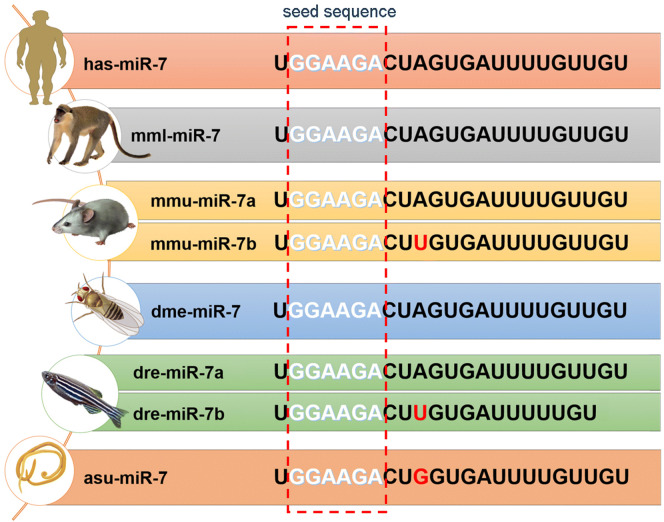
Homologous sequences of mature miR-7 in different species. The mature sequence of miR-7 has a perfectly conserved type in many species, including *Homo sapiens* miR-7 (has-miR-7), *Macaca mulatta* miR-7 (mml-miR-7), *Mus musculus* miR-7 (mmu-miR-7), *Drosophila melanogaster* miR-7 (dme-miR-7), *Danio rerio* miR-7 (dre-miR-7), *Asacris suum* miR-7 (asu-miR-7). A change in one of the bases of miR-7b (highlighted in red) exists in several species. The red virtual box is the seed sequence of mature miR-7

MicroRNA-7 (miR-7), as a unique member of the miRNAs family, has a high degree of conserved mature sequences with a length of 21–23 nt in different species (Fig. 1). In humans, three genes, including *miR*-*7*-*1*, *miR*-*7*-*2* and *miR*-*7*-*3* located on different chromosomes, respectively encode the corresponding precursor sequence which is processed and sheared into the same mature miR-7 sequence [1]. Similarly, in mice, *miR*-*7a*-*1*, *miR*-*7a*-*2* and *miR*-*7b* genes are also located on three different chromosomes and encode the corresponding precursor sequence which is eventually spliced into mature miR-7. Currently, the relevant studies on miR-7 molecule mainly focus on oncology. For example, Kabir et al. reported that miR-7 regulated the growth and invasion of sorafenib-resistant cells in human hepatocellular carcinoma through targeting TYRO3 [2]; Zhao et al. found that miR-7 prevented gastric cancer cell proliferation and tumorigenesis via repressing Nuclear Factor NF-Kappa-B P65 Subunit (P65) and Fos Proto-Oncogene (FOS, AP-1 Transcription Factor Subunit) expression [3]. Rodríguez-Antolín et al. showed that miR-7 methylation was a biomarker to predict poor survival in early-stage non-small cell lung cancer patients [4]. Our previous studies also showed that miR-7 overexpression could reduce the proliferation and metastatic capacity of human lung cancer cells in vitro and in vivo [5–8]. Moreover, the downregulation of miR-7 was closely related to the site mutation of the promoter region, which was correlated with poor prognosis of human lung cancer patients [9]. These foundings indicate that miR-7 might be used as an important potential target molecule for tumor diagnosis and treatment. Significantly, many recent studies have shown that miR-7 is highly enriched in brain tissue and is closely related to physiological and pathological process of brain [10–17], suggesting that it plays an important novel role in brain tissue development and disease occurrence, hence, it is may be a new promising therapeutic target for brain diseases.

## MiR-7 expression in brain

### MiR-7 distribution in brain

Many studies have shown that miR-7 and its precursors are expressed in the mammal lens [18], Nucleus accumbens (NACC) and suprachiasmatic nucleus (associated with the reward behavior and circadian rhythm of animals) [19] and neocortical and hippocampal regions [11]. However, the comprehensive data further show that the expression level of miR-7 and its precursor are higher in Pituitary and Hypothalamus [11, 20–22], while lower in Substantia nigra, Striatum, Cerebral cortex and Cerebellum [23]. Researchers speculate that this phenomenon may be related to miR-7-3 (the precursor of mature miR-7), which remains with the Pituitary specific factor 1 (PIT1) gene intron sequence [24]. These data suggest that miR-7 may play distinct roles in different brain regions.

The expression pattern of miR-7 in human brain tissue is also found in Mice [16], Rats [18], Cattle [20], Zebrafish [11], Amphioxus [25] and other species [26, 27]. For example, in Mice, miR-7 is also expressed in neurons with sensory or neurosecretory functions in the Hypothalamus [11, 16, 28]. Furthermore, in Amphioxus, miR-7 is expressed not only in the central nervous system, but also in the most anterior end of the pharyngeal endoderm at the later stage of neuronal development (20–22 h after fertilization) [25]. Moreover, other studies have shown that, in Zebrafish and Medaka, miR-7 has a conserved and highly restricted expression in the Medial forebrain at differentiation stages [29]; Besides, miR-7 is also restricted to be expressed in the developing forebrain with the prohormone vasotocin (vasopressin/oxytocin)-neurophysin from Annelids worms and Zebrafish. Importantly, the cell types characterized with dual sensory-neurosecretory properties in the forebrain are the starting point for the evolution of neurosecretory brain centers in Bilateria [11]. These data suggest that miR-7 expression is closely related with the sensory or/and neurosecretory neurons.

### The regulatory mechanisms of miR-7 expression

The expression level of mature miR-7 is affected by a variety of factors, including the direct or indirect regulation on the precursor sequence and mature miR-7. At the level of gene transcription: studies have shown that transcription factors Homeobox D10 (HOXD10) [30] and c-Myc [31] can bind to different parts of the pri-miR-7-1 promoter core sequence respectively to regulate the level of mature miR-7 (the region of c-Myc binding sequence is  − 539 to − 534; The binding regions of HOXD10 are − 1028 to − 1019 and − 968 to − 958). In addition, *miR*-*7*-*1* is the intron of the Heterogeneous Nuclear Ribonucleoprotein K (HNRNPK) gene, and *miR*-*7*-*3* is the intron of PIT1 gene [16]. As an intron miRNAs molecule, the expression level of miR-7 is also affected by the self-expression regulation mechanism of the host genes. For example, the expression of HNRNPK and PIT1 increases or decreases by the host under the action of certain physiological development and diseases, the expression levels of pri-miR-7-1 and pri-miR-7-3 will also be changed correspondingly, and then the level of mature miR-7 is affected inevitably [16].

In terms of the expression level of primary sequence and precursor sequence: Choudhury et al. found that the level of mature miR-7 enriched in specific brain regions was not consistent with the corresponding primary sequence expression [16], suggesting that there was a relevant regulatory mechanism during the process of the primary sequence of miR-7 (pri-miR-7) to the precursor sequence of miR-7 (pre-miR-7). Similarly, Kumar et al. found that Oleic Acid (OA) could prevent RNA recognition base sequence protein, namely Musashi homologous body 2/human HuR protein, from binding pri-miR-7-1 conservative terminal ring parts [32]. Thus, this process interferes with the formation of pre-miR-7-1 (precursor sequence of miR-7-1), resulting in the inconsistency between the expression of pri-miR-7 sequence and the expression of mature miR-7 sequence.

**Fig. 2:**
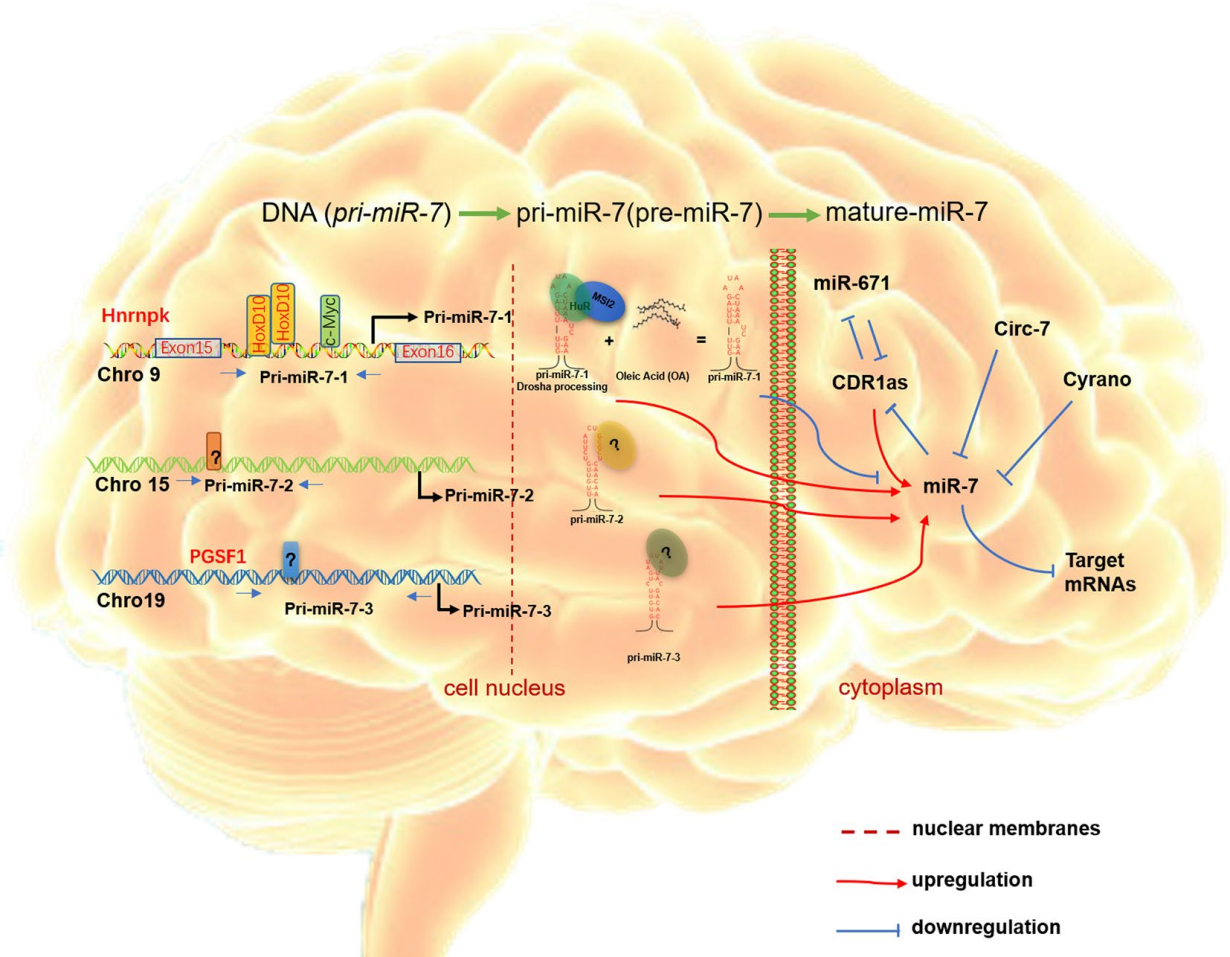
Schematic diagram of the regulatory mechanism of miR-7 expression. MiR-7 is transcribed from three different genomic loci on chromosomes 9, 15, 19 into primary miR-7 transcripts (pri-miR-7-1, pre-miR-7-2, pri-miR-7-3 respectively), which are processed into hairpin precursor molecules pre-miR-7, and then further into the same mature miR-7 sequences. There are always some molecules which affect miR-7 transcription involved in this process. Mature miR-7 sequence can target mRNAs to repress their expression, on the contrary, other antisense sequences interfere with miR-7 level

In addition, multiple regulatory mechanisms also exist at the mature level of miR-7. Studies have found that long non-coding RNA-circR-7 contains about 70 binding sites of miR-7 mature sequence and can effectively interfere with the level of mature miR-7 in a variety of tissue cells [13, 33]. Meanwhile, another long non-coding RNA-Cyrano (*linc*-*oip5*, *1700020I14Rik*) also has a conserved binding site to mature miR-7 sequence, which can directly bind and reduce the level of mature miR-7, and indirectly regulate the expression of miR-7 target molecules [10, 34]. Interestingly, recent studies also showed that Cerebellar Degeneration Related protein 1 antisense transcription (CDR1as, a complementary antisense sequence of miR-7), as a circular RNA highly expressed in brain neurons cell bodies, neurons axons and retina, could maintain miR-7 expression stability and positively regulate miR-7 level to ensure its regulation effect in neuronal cell activity [10, 12]. In addition, other members of the miRNA family, such as miR-671 also can negatively regulate the level of CDR1as and indirectly affect miR-7 expression [12, 35, 36]. These studies indicate that the regulatory mechanism of mature miR-7 expression is relatively complex (Fig. 2).

## The role of miR-7 in physiological function of brain

### Pituitary gland

Just as we have mentioned above, compared with other regions of the brain, the expression level of miR-7 is higher in the Pituitary gland, which is the most important endocrine gland in the body to regulate hormone secretion, indicating that miR-7 plays an important regulatory role in the process of pituitary hormone secretion. For example, Yuan et al. showed that miR-7 could directly bind to Prostaglandin F2 receptor negative regulator (PTGFRN), and then inhibit the expression of Prostaglandin F2 Receptor (PTGFR), thereby affecting the uterine contraction, ovulation, embryo implantation and other vital reproductive processes [20]. Recently, Ahmed et al. further reported that the effect of miR-7 expression on the Hypothalamus-Pituitary gonad axis. Their studies have shown that, in male and female mice, *miR*-*7a*-*2* gene deletion, following lower level of miR-7, leads to a low level of gonadotropin and sex steroid hormone, small testicular or ovarian, impaired sperm production, and lack of ovulation, resulting in infertility respectively. But when *miR*-*7*-*2* overexpression in the Pituitary gland, a raised miR-7 level can inhibit Golgi glycoprotein 1 (GLG1) expression and downstream bone morphogenetic protein 4 (BMP4) signaling pathway, and then reduce the level of PTGFRN and secretion of both Follicle-stimulating hormone (FSH) and Luteinizing Hormone (LH) [37]. These results suggest that the *miR*-*7a*-*2*/miR-7 axis regulates the secretion of FSH and LH by regulating the pituitary prostaglandin production and BMP4 signaling pathway, ultimately affects sexual maturity and reproductive function.

### Optic nervous system

MiR-7 also has an important regulating role in the visual system [18, 38–41]. Medullary neurons, as the largest visual processing center of the Drosophila brain, are derived from a sheet of neuroepithelial cells. During larval development, a wave of differentiation sweeps across the neuroepithelium, converting neuroepithelial cells into neuroblasts that sequentially express transcription factors specifying different neuronal cell fates. The switch from neuroepithelial cells to neuroblasts is controlled by a complex gene regulatory network. Caygill et al. found that during this transformation process, the expression level of miR-7 in neuroepithelial cells gradually increased, and the continuously up-regulated miR-7 promoted the stable transformation of neuroepithelial cells into neuroblasts by targeting Notch effector [39]. It is worth noting that miR-7 plays a key buffer and regulatory role in this process to ensure that a precise and rigorous transformation process which can be also maintained even under conditions of environmental stress, echoing the role of miR-7 in the eye imaginal disc [39]. Subsequent studies have shown that miR-7 expression is activated when progenitor cells begin to differentiate into photoreceptor cells, the process that is dependent on the EGF receptor (EGFR) signal which triggers ERK-mediated degradation of transcription factor Yan (a retinal cell differentiation inhibitor). In progenitor cells, Yan can prevent the transcription of miR-7, meanwhile miR-7 can bind with Yan mRNA 3′UTR sequences to inhibit Yan protein expression in the photoreceptor cells. These data have shown that Yan interacts with miR-7 to form a strict feedback effect model, in which Yan is highly expressed in the progenitor cells, while the miR-7 level is higher in the photoreceptor cells; when the EGFR signal shortly triggers Yan degradation in progenitor cells, companied with gradually increased miR-7 level to ensure accurate differentiation of photoreceptor cells [42], indicating that the spatiotemporal expression patterns of Yan and miR-7 are difference. In addition, human Paired box 6 (PAX6) is an important mediator of ocular development and formation. Needhamsen et al. further showed that miR-7 could inhibit the expression of PAX6 protein by directly binding with the 3′UTR of PAX6 [43]. These above studies have proved that miR-7 plays a key regulatory role in the development and formation of visual nerve cells through a sophisticated feedback regulatory network. However, whether miR-7 is involved in the functional regulation of visual neurons remains to be clarified.

### Cerebral cortex

Studies have found that, similar to photoreceptor cells, the expression level of miR-7 increases during the differentiation of cerebral cortical nerve cells or the development of Cortex after birth. However, compared with early differentiation of embryonic stem cells into nerve cells in mice, in the early development of cortex cell differentiation, miR-7 level is lower. However, in the seventh day of differentiation, 60–80% of the cells express higher level of miR-7 and neural precursor cells markers (CD57 and SOX1), suggested that miR-7 is closely related to the development of cerebral cortex cells [44]. Subsequent studies have showed downregulated expression of miR-7 in the mouse embryo cortical cells by miRNA-Sponge technology results in brain defect in mice. The mechanism is that when miR-7 is reduced, its target genes related to the p53 pathway, such as the upregulation of adenylate kinase 1 (AK1) and Cyclin dependent kinase inhibitor 1a (Cdkn1a), affects the normal transmission of p53 pathway and leads to the decreased production of cortical progenitor cells during brain development [45]. In addition, Sarangdhar et al. also found that the reduction of miR-7 level during embryonic development resulted in the change of brain morphology of mouse embryos, which was related to the increased expression of Cyrano, a lncRNA that is conservative expression in fertilized egg cells but closely related to brain development. Conversely, lowering the level of Cyrano can increase miR-7 level to improve the morphological abnormalities of fetal brain [34]. Similar studies have also shown that the loss of GLI Family Zinc Finger 3 (GLI3) in mouse embryonic cortex can increase the proportion of neural progenitor cells and newborn neurons in the brain, which resulting in the brain enlargement after birth and the abnormal folding structure of cortical midline. However, altered expression of miR-7 can restore the abnormal brain morphology in mice, as well as the production and migration of neurons. This mechanism is related to the up-regulation of the target molecule PAX6 expression after miR-7 change [46]. However, the relationship between PAX6 and GLI3 remains unclear. In conclusion, it has been proved that miR-7 is a stabilizer of these complex chain feedback and circulatory regulatory network, which is critical for stabilizing gene expression and determining cell fate. However, the complex molecular mechanism of its regulation needs to be further explored.

### Other brain functions

**Fig. 3:**
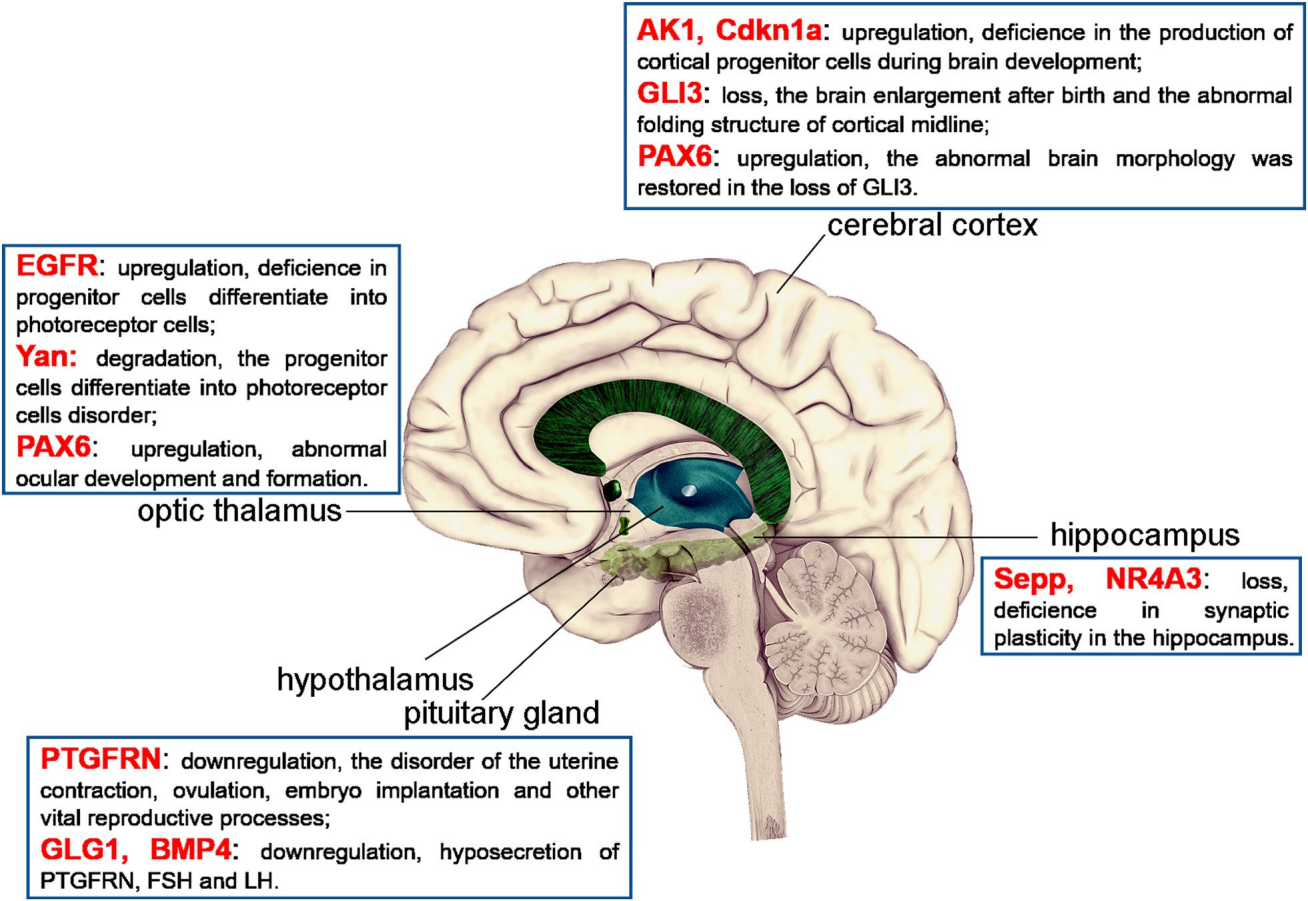
The targets of miR-7 and their function in physiological function of brain. The biological function of miR-7 target molecules in the Pituitary gland, Hypothalamus, Optic thalamus, Cerebral cortex and Hippocampus, respectively. *PTGFRN* prostaglandin F2 receptor negative regulator, *GLG1* golgi glycoprotein 1, *BMP4* bone morphogenetic protein 4, *FSH* follicle-stimulating hormone, *EGFR* EGF receptor, *PAX6* paired box 6, *AK1* adenylate kinase 1, *Cdkn1a* cyclin dependent kinase inhibitor 1a, *GLI3* GLI family zinc finger 3, *Sepp* selenoprotein P, *NR4A3* Nuclear Receptor Subfamily 4 Group A Member 3

The enriched expression of miR-7 in brain regions indicates that it may have other potentially unknown important roles in the brain. Such as Li et al. have shown that miR-7 is involved in the development and functional maintenance of various receptors in the body [41]. Recent studies have shown that miR-7 can enhance the formation of neurons in the subventricular region by inhibiting the Nod-like Receptor Protein 3 (NLRP3)/Caspase-1 pathway in human stem cells, and has a nice repair effect on neurons [47]. Other studies have also shown that miR-7 can be involved in synaptic plasticity in the Hippocampus through targeting the expression of Selenoprotein P (Sepp) [48] and Nuclear Receptor Subfamily 4 Group A Member 3 (NR4A3) [49, 50]. In addition, in Drosophila, mutations in miR-7 sequence do not cause a difference in appearance, but lead to abnormalities in sensory structures in stressful conditions [42, 44]. These researches indicate the potential role of miR-7 in physiological process of brain, which remains to be fully illuminated (Fig. 3).

## The role of miR-7 in brain diseases

**Fig. 4:**
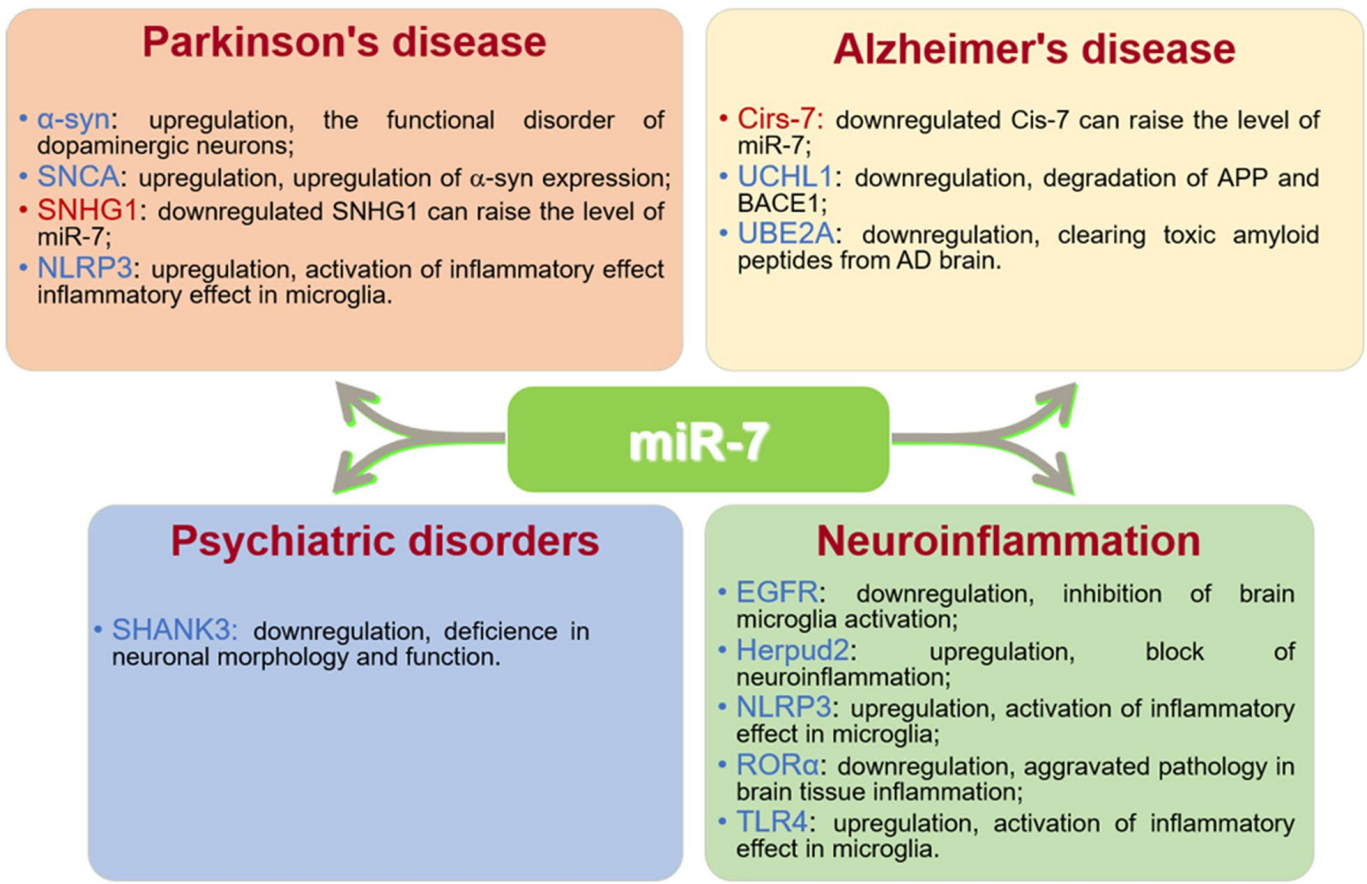
The targets of miR-7 and their function in brain diseases. The effects of miR-7 target molecules in the brain diseases including Parkinson’s disease, Alzheimer’s disease, Psychiatric disorders and Neuroinflammation respectively. The genes highlighted in red are the antisense interfering gene of miR-7. *PD* Parkinson’s disease, *AD* Alzheimer’s disease, *α*-*syn* α-synuclein, *SNCA* synuclein alpha, *SNHG1* small nucleolar RNA host gene 1, *NLRP3* nod-like receptor protein 3, *Aβ* beta-amyloid proteins, *BACE1* beta secretase 1, *APP* amyloid beta precursor protein, *UCHL1* ubiquitin carboxyl‐terminal hydrolase L1, *UBE2A* ubiquitin conjugating enzyme E2 A, *SHANK3* SH3 and multiple ankyrin repeat 3 domains

Accumulating evidence has shown that abnormal expression of miR-7 is involved in the development of various brain diseases, indicating miR-7 has an important role in pathological processes of brain (Fig. 4).

### Neurodegenerative diseases

#### Parkinson’s disease

The incidence of Parkinson’s disease (PD) accounts for 1% in people over 65 years old. The main characteristics of PD is the gradual loss of dopaminergic neurons in the Substantia nigra, in which abnormal expression of α-synuclein (α-syn) gathered in the form of fiber aggregates in the Substantia nigra and then mediates neurotoxicity to damage the function of dopaminergic neurons [51]. Studies have shown that the level of miR-7 in brain tissues of PD patients and PD animal models decreased significantly [52]. Currently, the mechanism of miR-7 is mainly focused on its regulatory effect on α-syn through multiple ways. Some studies have shown that the lack of miR-7 in brain tissues of PD patients is closely related to the accumulation of α-syn and the loss of dopaminergic neurons in the Substantia nigra, as well as the decreased dopamine secretion in the Striatum [53–55]. Choi et al. reported that miR-7 could accelerate the clearance of α-syn and its polymers, thereby promoting differentiation of ReNcell VM cells (human neural precursor cells) [55]. Junn et al. further found that miR-7 could inhibit α-syn protein level by directly acting on the 3′UTR of α-syn mRNA, thus protecting neurons from oxidative stress and proteasomal damage in MTPT-induced in vitro PD neurotoxin cell model and mouse model [23]. Meanwhile, other studies have shown that miR-7 can bind to the Synuclein Alpha (SNCA) 3′UTR of the Amyloid Precursor and then modulate the expression of α-syn [54]. In addition, miR-7 has been shown to promote the degradation of extracellular synthetic fibers of α-syn [55].

Microglia mediated inflammation reaction is involved in pathogenesis of PD [56, 57]. Studies have shown that miR-7 might regulate the activation of microglia, thereby controlling pathogenesis of PD. For example, in PD patients and dopaminergic SH SY5Y cells treated with MPPT, the expression of Small Nucleolar RNA Host Gene 1 (SNHG1) is increased, while miR-7 expression is decreased. Interestingly, downregulated SNHG1 in vitro can raise the level of miR-7 and inhibit LPS-induced BV2 microglial cells activation and inflammatory effect, and then prevent a potential loss of dopaminergic neurons in the Substantia nigra. Mechanistically, miR-7, as a regulator of SNHG1/NLRP3 axis, has an important regulating role in the inflammatory effect of microglia [58]. Besides, miR-7 can also inhibit the NF-κB signaling pathways to protect neurons from MTPT-induced cytotoxicity (dopaminergic SH SY5Y cells, human neural progenitor cells ReNcell VM cells and primary neurons in mice) [59]. In addition, miR-7 can restore anti-apoptotic protein molecule BCL2 level by inhibiting mTOR signaling pathway, thus protecting the neuron cells damage induced by MTPT in PD model [60]. These studies suggest that miR-7 has a great potential role in the pathogenesis of PD though regulating multiple cells and molecules.

#### Alzheimer’s disease

Recently, miR-7 has also been found to play an important regulatory role in the development of Alzheimer’s disease (AD). For example, Puthiyedth et al. analyzed the differentially expressed genes in different brain regions, including the Entorhinal Cortex, Hippocampus, Middle temporal gyrus, Posterior cingulate cortex, Superior frontal gyrus and visual cortex brain regions, from 161 clinical brain tissue samples (74 non-demented controls, 87 AD). They found that miR-7-1, a precursor of miR-7, was significantly upregulated in the all regions of brain tissue from AD patients, suggesting that miR-7 was closely related to the occurrence of AD [61]. The abnormal accumulation of beta-amyloid proteins (Aβ) in the brain is a key feature of AD progression. Beta Secretase 1 (BACE1) is a rate-limiting enzyme that forms Aβ and Amyloid beta Precursor Protein (APP) which is a precursor that forms Aβ. Shi et al. showed that Cirs-7, a regulator of BACE1 and APP expression, was significantly down-regulated in the brain tissue of AD patients [62]. Further studies have shown that low CDR1as level can lead to the increased expression of miR-7 which downregulates the activity of ubiquitin conjugating enzyme E2 A (UBE2A), thereby resulting in impaired clearing of toxic amyloid peptides from brain in AD, suggesting that miR-7 may be a new target for AD treatment [36, 63].

### Psychiatric disorders

The abnormal miR-7 expression has been also linked with a neurological disorder called Schizophrenia which affects about 1 percent of adults worldwide and has the greatest impact on quality of life compared to other neurological disorders. However, the exact etiology of Schizophrenia is still unknown. Studies have shown that miR-7 expression is up-regulated in the plasma and the frontal lobe of brain in patients with Schizophrenia; Moreover, the effect of miR-7 on Schizophrenia might be linked to directly target SH3 and Multiple Ankyrin Repeat 3 Domains (SHANK3) which is closely related to synaptic plasticity and memory [64–66]. Furthermore, the abnormal level of miR-7 in Schizophrenia is associated with its primary sequence *pri*-*miR*-*7*-*3* [67]. These findings indicate that miR-7 might be involved in the development of Schizophrenia, which still need to be further illustrated.

### Neuroinflammation

It is well known that inflammation is a common pathological basis for various neurological diseases. Recently, a large number of studies have shown that miR-7 is involved in the occurrence of neuroinflammation [58, 68]. Such as, Cao’s study found that long noncoding RNA SNHG1 promoted neuroinflammation in the pathogenesis of PD via modulating miR-7/NLRP3 pathway [58]; Dong et al. also proved that miR-7 could target the 3′UTR of Herpud2 which encoded Endoplasmic Reticulum (ER) stress protein-HERP2, indicating miR-7-targeted ER stress acted as a molecular brake on neuroinflammation [68]. Furthermore, Zhang et al. showed that miR-7 could inhibit EGFR/STAT3 pathway and TLR4 expression to block brain microglia activation, cytokine production and slow the secondary damage of brain tissue in a Cerebral Hemorrhage model [69]. Separately, in our recent study, we found that miR-7 could govern the pathology of brain tissue inflammation through controlling the inflammatory reaction of neuronal cells in brain tissue inflammation model (BTI) [70]. Therefore, these current findings might highlight not only the important role of miR-7 but also the relationship among neurons and other cells such as microglia in pathogenesis of neuroinflammation.

### Other brain diseases

MiR-7 also plays an important regulatory role in other brain diseases. For instance, Nelson et al. found that there was abnormal expression of miR-7 in brain tissue from the patients with Lewy body dementia (DLB), which is characterized by fluctuating cognitive dysfunction, visual hallucinations and Parkinson’s disease syndrome [71]. Moreover, other studies have shown that miR-7 is significantly increased in the peripheral blood of patients with Cerebral artery malformation and is involved in the pathological development of Cerebral artery malformation through targeting VEGF pathway [72]. Recently, Lee et al. also found that the serum expression level of miR-7-5p was significantly increased in Bipolar II disorder patients [73]. Besides, miR-7 expression in peripheral blood from patients with Acute ischemic stroke is higher than healthy controls [74]. Furthermore, inhibition of miR-7 level, in the process of ischemic postconditioning, can prevent mitochondria damage and restore the ATP activity, thereby improving neuron function after focal ischemia, suggesting miR-7 may be a potential therapeutic target in Acute ischemic stroke [74].

## Conclusions

**Fig. 5:**
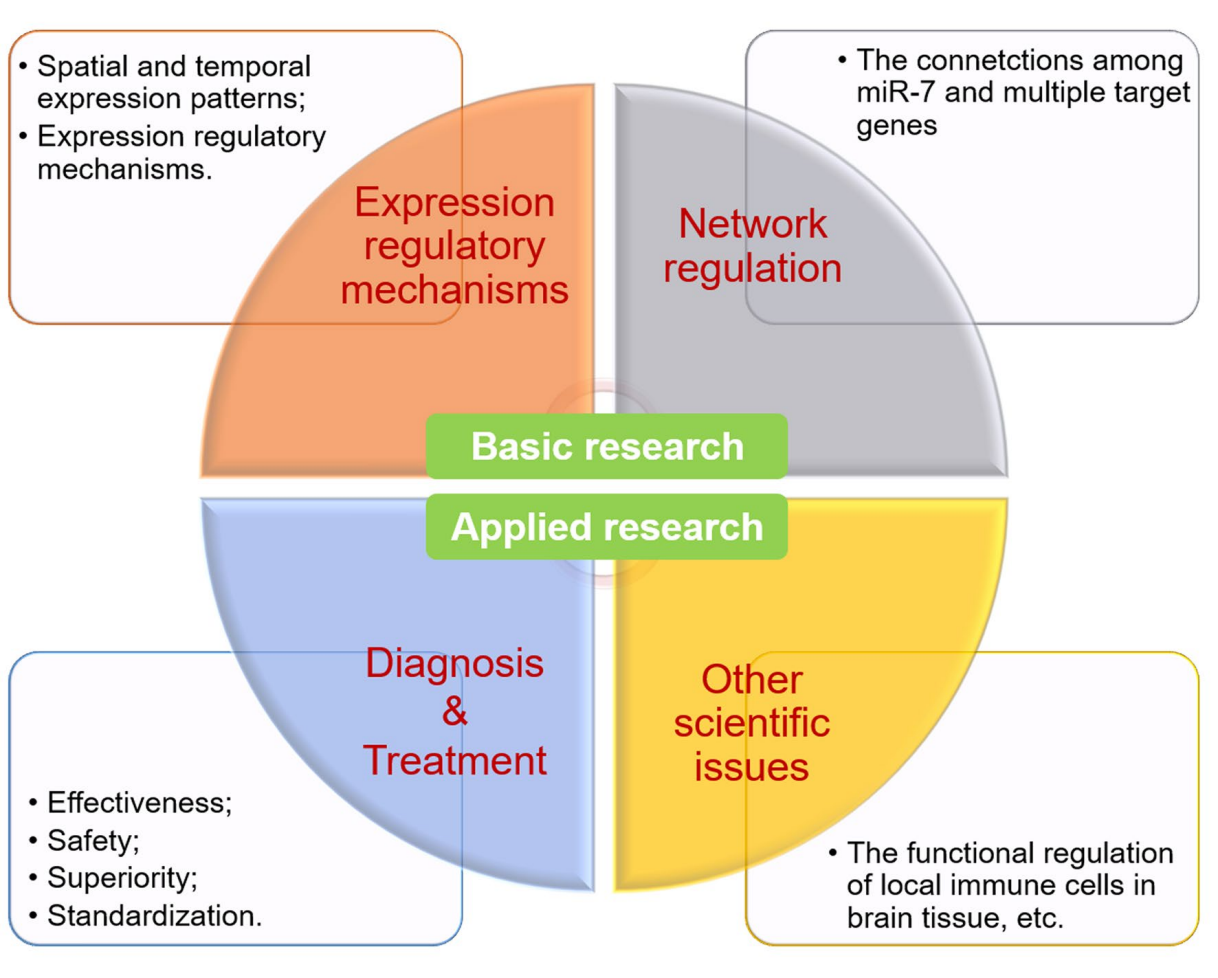
A sketch of scientific issues of miR-7 in the physiological and pathological processes of brain. Currently, there are still many unclear scientific issues, belongs to basic and applied research fields, on the role miR-7 in the development and diseases of brain

Up to now, the important progression on biological role of miR-7 in brain development and diseases has been reached. Among all of miRNA family members, miR-7 is dominantly expressed in brain tissue and plays important roles in the development of brain tissue and the progression of brain diseases (Table 1), suggesting that miR-7 may be a promising novel intrinsic regulator for brain development and disease occurrence. Alongside, many important scientific issues, at least three major aspects, still need to be further illustrated in the future (Fig. 5). Firstly, what are the spatial and temporal expression patterns of miR-7 and their regulatory mechanisms in the physiological and pathological processes of brain? Especially, the expression patterns of miR-7 in different types of cells in the process of brain development and disease occurrence are still largely unknown. Secondly, the current knowledge thus far on the underlying interactions among miR-7 and its target molecules in brain development and diseases are still limited. Interestingly, in our most recent work, we found that miR-7 synergizes with, but not antagonizes, its target RORα to control the pathology of BTI [70], indicating the complexity of network among miRNAs and their targets in biological process. Therefore, what are the exact connections among miR-7 and its multiple targets in distinct types of cells in different brain diseases? Thirdly, the potential value of miR-7 in the diagnosis and therapeutic strategies of brain diseases need to be further clarify. For instance, the expression level of miR-7 is variable in different brain diseases. We propose it reflects the complexity of the role of miR-7, especially in distinct regions of brain, in different brain diseases. Therefore, the combination of miR-7 expression and other factors, including clinical characters, of different diseases might be much valuable for the application of miR-7 as a bio-maker in diagnosis of brain diseases.

**Table 1:** The effects of miR-7 target in physiological and pathological processes of brain

Targets	Brain regions	Expression	Effects	Refs.
PTGFRN	Pituitary gland	Downregulation	lower level of PTGFR and LH release; the decrease of uterine contraction, ovulation, embryo implantation and other vital reproductive processes	[20]
GLG1, BMP4	Pituitary gland	Downregulation	The lower secretion of PTGFRN, FSH and LH	[37]
Notch effector	Optic nerve epithelial cells	Downregulation	The more stable transformation of neuroepithelial cells to neuroblastocytes	[39]
Yan	Visual progenitor cell	Downregulation	The inhibition of miR-7 transcription, ensure that photoreceptors are successfully and accurately differentiated	[42]
PAX6	Eye and brain	Downregulation	The maintenance of ocular development and formation	[43]
Ak1, Cdkn1a	Embryo cortical	Upregulation	Decreased production of cortical progenitor cells during brain development	[45]
GLI3	Embryonic cortex	Loss	The increased proportion of neural progenitor cells and newborn neurons in the brain, which resulting in the brain enlargement after birth and the abnormal folding structure of cortical midline	[46]
Sepp, NR4A3	Hippocampus	Loss	Deficience in the synaptic plasticity in the hippocampus	[48, 49]
α-syn	Substantia nigra	Upregulation	Increased neurons from oxidative stress and proteasomal damage,	[23]
SNCA	Substantia nigra	Upregulation	The increased expression of α-syn	[54]
mTOR	Substantia nigra	Downregulation	Protecting the neuron cells damage	[60]
SHANK3	Cerebral cortex	Downregulation	Deficience in synaptic plasticity and relation with memory and learning	[66]
Herpud2	Brain tissue	Upregulation	Brake on neuroinflammation	[68]
TLR4	Microglia	Downregulation	Block of brain microglia activation, cytokine production and slow the secondary damage of brain tissue	[69]
RORα	Neuron	Downregulation	aggravated pathology of brain tissue inflammation	[70]

*PTGFRN* Prostaglandin F2 receptor negative regulator; *PTGFR* prostaglandin F2 receptor; *LH* luteinizing hormone; *GLG1* golgi glycoprotein 1; *BMP4* bone morphogenetic protein 4; *FSH* follicle-stimulating hormone; *PAX6* paired box 6; *AK1* adenylate kinase 1; *Cdkn1a* cyclin dependent kinase inhibitor 1a; *GLI3* GLI family zinc finger 3; *Sepp* selenoprotein P; *NR4A3* Nuclear Receptor Subfamily 4 Group A Member 3; *α*-*syn* α-synuclein; *SNCA* synuclein alpha; *SHANK3* SH3 and multiple ankyrin repeat 3 domains

In all, the in-depth studies on the expression patterns of miR-7, the networks among miR-7 and its targets, and the role of miR-7 in different types of cells, as well as the prognostic and therapeutic value of miR-7 in brain diseases, will undoubtedly throw a new light on the knowledge on biological roles of miRNAs in physiological and pathological process of brain and ultimately benefit clinical outcome of patients with brain diseases.

## Data Availability

Not applicable.

## Abbreviations

miR-7: MicroRNA-7
P65: Nuclear Factor NF-Kappa-B P65 Subunit
FOS: Fos proto-oncogene
NACC: Nucleus accumbens
PIT1: Pituitary specific factor 1
HOXD10: Homeobox D10
HNRNPK: Heterogeneous nuclear ribonucleoprotein K
pri-miR-7: Primary sequence of miR-7
pre-miR-7: Precursor sequence of miR-7
MSI2: Musashi homologous body 2
HuR: Hu antigen R
pre-miR-7-1: Precursor sequence of miR-7-1
CDR1as: Cerebellar degeneration related protein 1 antisense transcription
PTGFRN: Prostaglandin F2 receptor negative regulator
PTGFR: Prostaglandin F2 receptor
LH: Luteinizing hormone
GLG1: Golgi glycoprotein 1
BMP4: Bone morphogenetic protein 4
FSH: Follicle-stimulating hormone
EGFR: EGF receptor
PAX6: Paired box 6
AK1: Adenylate kinase 1
Cdkn1a: Cyclin dependent kinase inhibitor 1a
GLI3: GLI family zinc finger 3
Sepp: Selenoprotein P
NR4A3: Nuclear Receptor Subfamily 4 Group A Member 3
α-syn: α-synuclein
SNCA: Synuclein alpha
SNHG1: Small nucleolar RNA host gene 1
NLRP3: Nod-like receptor protein 3
PD: Parkinson’s disease
AD: Alzheimer’s disease
Aβ: Beta-amyloid proteins
BACE1: Beta secretase 1
APP: Amyloid beta precursor protein
UCHL1: Ubiquitin carboxyl‐terminal hydrolase L1
UBE2A: Ubiquitin conjugating enzyme E2 A
SHANK3: SH3 and multiple ankyrin repeat 3 domains
ER: Endoplasmic reticulum
BTI: Brain tissue inflammation model
DLB: Lewy Body Dementia
